# Clinical Efficacy of Standardized Care for Abdominal Drainage Tubes in the Treatment of Acute Appendicitis: A Single‐Center Retrospective Cohort Study

**DOI:** 10.1155/emmi/7131025

**Published:** 2026-07-22

**Authors:** Jiamei Lin, Yanan Zhou, Zipeng Xu, Wenhui Shi, Yan Huang, Genxi Tong, Fengjun Cai, Qinyan Yang, Chaobo Chen

**Affiliations:** ^1^ Department of General Surgery, Xishan People’s Hospital of Wuxi City, Wuxi 214105, China

**Keywords:** acute appendicitis, AD tube, LA, nursing, retrospective study, standardization

## Abstract

**Objective:**

Although abdominal drainage (AD) tubes have become an effective means to prevent and observe complications after laparoscopic appendectomy (LA), there are still controversies, and no standardized nursing process (SNP) is shown for the management of drainage tube care. This investigation aims to assess the effectiveness of SNP management in AD tubes after LA.

**Methods:**

This retrospective cohort investigation comprised individuals who had LA surgery with abdominal drain placement for acute appendicitis from July 2021 to June 2024. Individuals were classified into the SNP and non‐SNP groups. A comparison of the outcomes was conducted, while univariate and multivariate analyses were conducted to assess the factors related to the SNP. *p*‐value < 0.05 was regarded as significant.

**Results:**

A total of 367 patients were enrolled in this investigation, and no significant variations were shown in baseline characteristics between the SNP (*n* = 199) and non‐SNP groups (*n* = 168). Multivariable logistic regression analysis displayed that SNP management was an important factor in decreasing postoperative 24‐h pain scores (OR = 0.22, 95% CI: 0.12–0.42, *p* < 0.001), improving the out‐of‐bed activity rate (OR = 2.33, 95% CI: 1.37–3.96, *p* < 0.05) and patient’s degree of satisfaction after surgery (OR = 16.02, 95% CI: 9.06–28.32, *p* < 0.001). However, SNP had no significant effect on the incidence of postoperative drainage tube dislodgement (OR = 0.49, 95% CI: 0.16–1.51, *p* > 0.05).

**Conclusions:**

In this retrospective cohort, the implementation of SNP was associated with significantly lower postoperative 24‐h pain scores, a higher out‐of‐bed activity rate, and greater patient satisfaction after LA surgery. These findings suggest that SNP may be a beneficial component of postoperative care and warrant further investigation.

## 1. Introduction

Acute appendicitis is a prevalent surgical emergency and a leading cause of acute abdomen in general surgery. Patients typically present to the emergency department, and prompt surgical intervention is the cornerstone of management. Appendectomy, including open appendectomy and laparoscopic appendectomy (LA), is the most effective and direct method for treating acute appendicitis [1]. Emergency appendectomy is chosen by about 90% of patients. It should be noted that about 7%–10% of patients will develop complications after appendectomy, mainly infection‐related, such as wound infection, abdominal abscess, and postoperative intestinal obstruction [2].

Currently, after appendectomy, using the prophylactic abdominal drainage (AD) tubes remains mainly depending on the surgeon’s preferences and experience and to some extent on the severity of appendicitis during surgery [3]. The main purpose is to lessen the occurrence of postoperative infectious complications [4]. The AD tube is an effective means of preventing complications after LA, but complications still affect patient prognosis [5]. Meanwhile, an increasing number of researchers have objected to this, arguing that the placement of AD tubes cannot lessen the occurrence of complications after an appendectomy but will elevate the prevalence of complications linked to the drainage tube [2, 6]. However, the researchers found no significant difference in the prevalence of complications between placing and not placing drainage tubes after appendectomy [4, 7]. Therefore, utilizing the AD tubes after appendectomy to decrease abdominal complications is still controversial [4, 8].

In fact, previous studies have focused more on whether to position an AD tube. However, few studies have focused on the standardized nursing process (SNP) for AD tubes in clinical practice. If postoperative drainage tube care is unreasonable, is it also a factor that affects patient prognosis and leads to drainage tube–related complications? There is no research report yet on developing an SNP for AD tubes. Should the focus be shifted towards how to care for AD tubes effectively? It is worth further exploring and researching.

This study assessed the effect of SNP management of drainage tubes in LA patients in a single center and observed the clinical role of AD tubes after appendectomy from the perspective of AD tube nursing management. We aim to examine the feasibility of SNPs by observing and analyzing the effect of standardized nursing on AD tubes after LA and to offer a theoretical foundation for the continuous improvement of subsequent nursing strategies.

## 2. Methods

### 2.1. Study Design and Patients

A retrospective cohort study of individuals who underwent LA with AD for acute appendicitis (July 2021–June 2024) was conducted. The SNP was formally implemented in January 2023. Patients operated before January 2023 received conventional (nonstandardized) nursing care and were assigned to the non‐SNP group (historical controls). Patients operated from January 2023 onward received care under the SNP protocol and were assigned to the SNP group. During the entire study period, there were no changes in analgesia protocols, enhanced recovery after surgery practices, nursing staffing, or surgical team composition. All operations were performed by the same experienced surgical team. The investigation was conducted as per the Declaration of Helsinki, and the Institutional Review Board of Xishan People’s Hospital gave its approval to it (Approval No. xs2025ky001). Informed consent was waived because of the retrospective design.

Inclusion criteria: (1) diagnosis of acute appendicitis (including acute suppurative appendicitis and acute suppurative appendicitis with perforation); (2) age 18–80 years; and (3) AD tube placement after LA (both SNP and non‐SNP groups).

Exclusion criteria: (1) conversion from LA to open surgery; (2) LA without drainage tube placement; (3) unmanageable coagulation disorders; (4) anesthesia intolerance; (5) pregnancy or breastfeeding; (6) loss to follow‐up; and (7) procedures performed by junior surgeons.

### 2.2. Surgical Technique

All surgeries were performed by the same surgical team using a standard three‐port LA technique as previously described [9, 10]. All procedures were conducted under the American Society of Anesthesiologists (ASA) classification [11].

### 2.3. Indications for Drain Placement

Drainage tubes were placed according to the attending surgeon’s intraoperative assessment, including perforation, abundant purulent exudate, uncertain hemostasis, or severe tissue edema.

To minimize selection bias, all surgeons followed the same intraoperative indications; baseline comparison confirmed no significant difference in appendicitis type between groups (*p* = 0.575); multivariate regression was adjusted for appendicitis type, ASA score, and inflammatory markers.

### 2.4. SNP

Herein, all individuals in the SNP group were uniformly provided with nursing‐feedback‐nursing by a professional nursing team based on the SNP management model.

**FIGURE 1:**
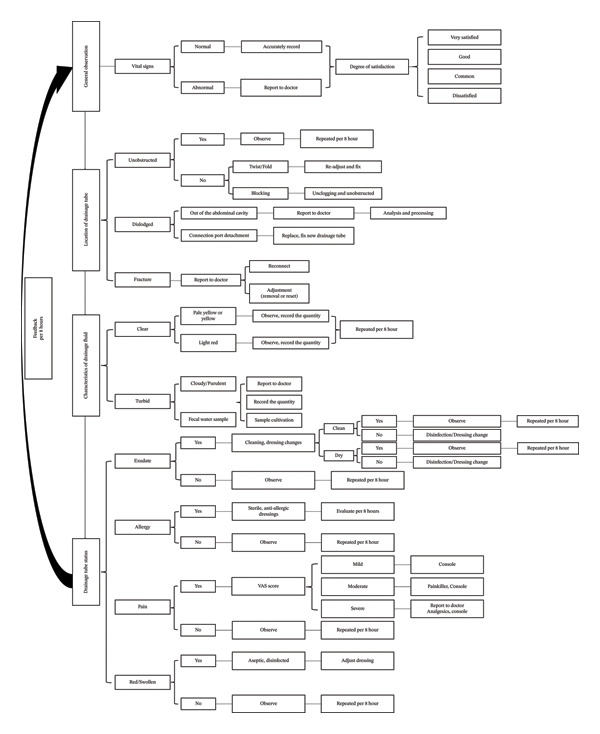
Flow‐chart of SNP. A representative process was facilitated by feedback loop management and nursing adjustments to the abdominal drainage after LA.

The SNP was implemented as a step‐by‐step, four‐stage cyclic protocol performed by a dedicated nursing team, with mandatory documentation every 8 h (Figure 1):

1. General observation: monitor vital signs every 8 h; assess tube‐related pain (VAS) and patient tolerance; record comfort and discomfort related to the tube.
2. Drainage tube positioning: confirm the insertion depth, fix the tube with a semi‐permeable dressing, mark the tube at the skin level to detect displacement ≥ 2 cm, keep the drainage bag below the wound level to prevent reflux, and adjust for kinking or compression.
3. Drainage fluid evaluation: measure the drainage volume every 8 h; classify fluid as transparent, serous, turbid, purulent, or bloody; notify the surgeon and send for culture if turbid/purulent.
4. Tube site and complication assessment: inspect the exit site for redness, swelling, exudate, or allergy; check dressing for leakage (dry/damp/saturated); document and notify the physician for abnormalities.

All data were reviewed by a senior nurse every 8 h, with immediate intervention for obstruction, dislodgement, or infection signs, and daily adjustment of care measures.

Before SNP implementation, all involved nurses completed a standardized training program including a 4‐h theoretical training, a 2‐h practical training, and a competency test with a passing score of ≥ 90. A monthly refresher training and audit were conducted to ensure consistency.

To monitor protocol adherence, a 10‐item standardized checklist was used for each patient in the SNP group. Each item was scored 10 points (total 100 points), and the adherence rate was calculated as (actual score/100) × 100%. The overall adherence rate was 96.2% (range: 92%–100%), confirming high implementation consistency.

### 2.5. Data Collection

Data collection and monitoring were conducted for the patients after the treatment of LA. Data comprised gender, age (year), fever peak (°C), BMI (body mass index), leukocyte (10 [9]/L), C‐reactive protein (CRP), neutrophil (%), direct bilirubin (DB), total bilirubin (TB), alanine transaminase (ALT), drainage tube placement time (Day), aspartate transaminase (AST), VAS score (postoperative 24 h), out‐of‐bed activity rate (24 h postoperatively, %) (No/Yes), volume of drainage (mL), drainage tube dislodged (No/Yes), drainage tube leakage (No/Yes), postextubation leakage (No/Yes), degree of satisfaction, diabetes (No/Yes), hypertension (No/Yes), American Society of Anesthesiologists (ASA) score (I/II/III), and type (purulent appendicitis/gangrenous appendicitis/perforated appendicitis). All patients were monitored for 3 months.

### 2.6. Definition

The VAS score was assessed at 24 h after surgery (±1 h) using a standard 10‐cm horizontal line, where 0 represented “no pain” and 10 represented “worst pain imaginable.” After a standardized explanation by a trained nurse, patients independently marked their pain level, and the nurse recorded the corresponding integer value (0–10).

Patient satisfaction was assessed at hospital discharge using an institution‐developed self‐reported questionnaire comprising 20 items, each rated on a 5‐point Likert scale (1 = strongly dissatisfied to 5 = strongly satisfied). The total score ranged from 20 to 100, with higher scores indicating greater satisfaction. For descriptive presentation, raw scores were categorized into a 4‐level ordinal scale: 81–100 (level 4, highly satisfied), 61–80 (level 3, satisfied), 41–60 (level 2, moderately satisfied), and 20–40 (level 1, dissatisfied). The questionnaire was developed based on the Patient Satisfaction With Nursing Care Quality Questionnaire (PSNCQQ), a validated instrument with excellent psychometric properties (Cronbach’s *α* = 0.97) for nursing quality improvement [12]. It was revised to fit the clinical scenario of AD care after LA. Content validity was evaluated by three senior surgical nurses and two surgeons, and construct validity was examined using exploratory factor analysis. The internal consistency of the questionnaire was evaluated using Cronbach’s αbased on the total study sample. The results showed good reliability (Cronbach’s *α* = 0.88 for the total sample; 0.86 for the non‐SNP group and 0.88 for the SNP group), indicating satisfactory internal consistency.

Drainage tube dislodged: unplanned outward displacement of the tube by ≥ 2 cm from its documented position at the skin entry site (skin entry site marking). This was assessed by nursing staff via measurement and confirmed by the surgical team.

Drainage tube leakage: drainage fluid leaking around the tube at its skin entry site, adjudicated based on dressing condition: yes—the first layer of dressing was damp or saturated, potentially soaking through to outer layers, requiring a dressing change; no—the site was dry or with only minor staining on the first layer.

Postextubation leakage: persistent drainage from the empty tube tract after removal, adjudicated similarly based on the dressing condition at the wound site: yes—damp or saturated dressing requiring continued care; no—dry or minimally stained.

### 2.7. Statistical Analysis

SPSS (v22.0) was utilized to conduct statistical analyses (IBM Corp., Armonk, USA). The Shapiro–Wilk test was utilized to assess the normality of continuous variables. Data with normal distribution are reported as mean ± standard deviation and compared using the independent *t*‐test, while data with abnormal distribution are reported as median (25^th^–75^th^ percentiles), and the Mann–Whitney *U* test was utilized for analysis. Categorical variables are shown as counts (percentages), and the chi‐squared or Fisher’s exact test was utilized for comparisons. Univariate and multivariate binary logistic regression analyses were conducted to detect independent factors related to outcomes [13]. Variables with extremely low event counts (≤ 10 events) that caused complete separation were excluded from logistic regression, in accordance with the events‐per‐variable principle [14]. For the logistic regression analyses, the VAS score was dichotomized based on the median value of the cohort (median = 3). Scores ≤ 3 were defined as better pain control, and scores ≥ 4 were defined as poorer pain control. Patient satisfaction was also dichotomized: level 4 (highly satisfied) was defined as the positive outcome, and levels 1–3 were combined as the reference group. *p*‐value < 0.05 was regarded as significance.

## 3. Results

### 3.1. Characteristics of the Patients

**FIGURE 2:**
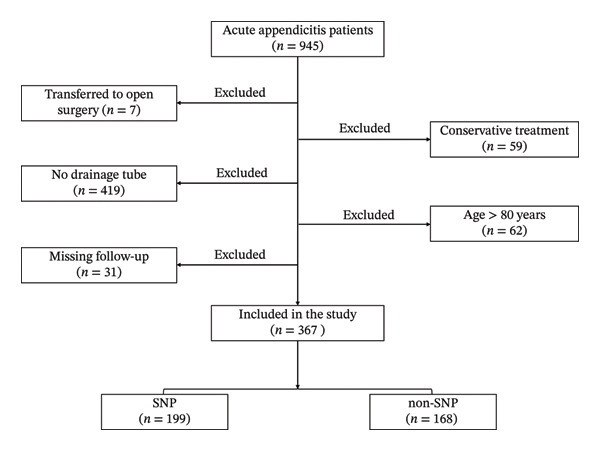
Detailed surgery flow diagram.

A total of 945 patients diagnosed with acute appendicitis at the Xishan people’s Hospital of Wuxi City between July 2021 and June 2024 were initially screened. Of these, 419 patients without a drainage tube and 59 who received conservative treatment were excluded. Additionally, seven patients converted to open surgery, 62 patients aged over 80 years, and 27 patients with missing follow‐up data were excluded. Ultimately, 367 patients were classified as 199 in the SNP group and 168 in the non‐SNP group (Figure 2).

Comparison between the non‐SNP and SNP groups showed no significant variations in sex, fever peak, age, BMI, leukocyte count, CRP, HBP, neutrophils, DB, TB, ALT, or AST (*p* > 0.05; Table 1). These findings indicated that SNP management was not associated with baseline inflammatory markers or liver function indices.

**TABLE 1:** Characteristic and clinical features of patients.

Item	Non‐SNP (*n* = 168)	SNP (*n* = 199)	*p* value
Gender			0.348
Female	62 (36.9%)	83 (41.71%)	
Male	106 (63.1%)	116 (58.29%)	
Age (year)	46 (32, 59)	44 (34, 59)	0.765
BMI	24.22 (21.48, 25.96)	23.78 (21.9, 26.17)	0.946
Fever peak (°C)	36.9 (36.7, 37.3)	37 (36.7, 37.4)	0.657
Leukocyte (10^9/L)	12.84 (10.26, 15.45)	12.23 (9.36, 14.72)	0.100
CRP	58.95 (23.98, 116.5)	51.1 (23.7, 86.3)	0.227
Neutrophil (%)	84.45 (77.57, 89)	84.2 (77.5, 89.5)	0.881
DB	5.3 (3.9, 6.93)	5.4 (3.9, 7.65)	0.577
TB	15.95 (12.17, 20.92)	17.3 (12.05, 22.8)	0.221
ALT	17 (11, 28)	16 (12, 24)	0.613
AST	16 (13, 22)	16 (13, 21)	0.395
Diabetes			0.289
No	153 (91.07%)	187 (93.97%)	
Yes	15 (8.93%)	12 (6.03%)	
Hypertension			0.131
No	155 (92.26%)	174 (87.44%)	
Yes	13 (7.74%)	25 (12.56%)	
ASA score			0.226
I	145 (86.31%)	164 (82.41%)	
II	19 (11.31%)	33 (16.58%)	
III	4 (2.38%)	2 (1.01%)	
Type			0.575
Purulent appendicitis	154 (91.67%)	183 (91.96%)	
Gangrenous appendicitis	7 (4.17%)	11 (5.53%)	
Perforated appendicitis	7 (4.17%)	5 (2.51%)	

*Note:* ALT, alanine transaminase; AST, aspartate transaminase.

Abbreviations: ASA, American Society of Anesthesiologists; CRP, C‐reactive protein; DB, direct bilirubin; TB, total bilirubin.

### 3.2. Comparison of Postoperative Indicators of LA Related to Drainage Tubes

However, by comparing the postoperative indicators of LA between these two groups, we found that the out‐of‐bed activity rate (24 h postoperatively, %) and degree of satisfaction in the SNP group showed a significant elevation compared to the non‐SNP group (*p* < 0.001, Table 2). At the same time, the VAS score (postoperative‐24‐h) and the probability of the drainage tube being dislodged were substantially lower (*p* < 0.05, Table 2).

**TABLE 2:** Analysis of drainage tube–related parameters after surgery.

Item	Non‐SNP (*n* = 168)	SNP (*n* = 199)	*p* value
VAS score (postoperative‐24‐h)			**< 0.001**
2	45 (26.79%)	100 (50.25%)	
3	80 (47.62%)	85 (42.71%)	
4	36 (21.43%)	12 (6.03%)	
5	7 (4.17%)	2 (1.01%)	
Out‐of‐bed activity rate (24 h postoperatively, %)			**0.001**
No	50 (29.76%)	30 (15.08%)	
Yes	118 (70.24%)	169 (84.92%)	
Drainage tube placement time (Day)	3 (2, 4)	2 (2, 3)	0.214
Volume of drainage	63 (32, 125.75)	55 (27.5, 103)	0.197
Drainage tube dislodged			**0.035**
No	156 (92.86%)	194 (97.49%)	
Yes	12 (7.14%)	5 (2.51%)	
Drainage tube leakage			0.502
No	168 (100%)	197 (98.99%)	
Yes	0 (0%)	2 (1.01%)	
Postextubation leakage			0.190
No	165 (98.21%)	199 (100%)	
Yes	3 (1.79%)	0 (0%)	
Degree of satisfaction			**< 0.001**
1	6 (3.57%)	0 (0%)	
2	21 (12.5%)	3 (1.51%)	
3	92 (54.76%)	24 (12.06%)	
4	49 (29.17%)	172 (86.43%)	

*Note:* Bold values indicates statistically significant differences (*p* < 0.05).

### 3.3. SNP Associated With Reducing Postoperative Pain Score

Furthermore, to explore the effect of SNP management on postoperative VAS score (postoperative‐24‐h), we performed a univariate logistic regression analysis, and the outcomes illustrated that SNP management could successfully reduce postoperative 24‐h pain score (OR = 0.22, 95% CI: 0.12–0.42). While multivariate regression analysis excluded confounding factors, SNP management was still confirmed as an important factor in reducing postoperative 24‐h pain scores (OR = 0.22, 95% CI: 0.12–0.42) (*p* < 0.001, Table 3).

**TABLE 3:** Univariable and multivariable analyses for VAS score (postoperative‐24‐h).

Items	Univariable		Multivariable	
	OR [95% CI]	*p* value	OR [95% CI]	*p* value
SNP	0.22 [0.12, 0.42]	**< 0.001**	0.22 [0.12, 0.42]	**< 0.001**
Gender	1.14 [0.64, 2.05]	0.654		
Age (year)	1.00 [0.99, 1.02]	0.712		
BMI	1.03 [0.95, 1.11]	0.520		
Fever peak (°C)	0.98 [0.80, 1.20]	0.826		
Leukocyte (10^9/L)	0.98 [0.92, 1.04]	0.511		
CRP	1.00 [1.00, 1.00]	0.904		
Neutrophil (%)	1.00 [0.98, 1.02]	0.942		
DB	0.99 [0.91, 1.09]	0.850		
TB	0.99 [0.95, 1.02]	0.397	0.99 [0.96, 1.02]	0.560
ALT	1.00 [0.99, 1.01]	0.847		
AST	1.00 [0.99, 1.01]	0.942		
Diabetes	1.15 [0.49, 2.72]	0.742		
Hypertension	0.61 [0.30, 1.28]	0.191		
ASA score				
I	1.00 [Reference]			
II	0.73 [0.38, 1.39]	0.338		
III	0.84 [0.17, 4.23]	0.831		
Type				
Purulent appendicitis	1.00 [Reference]			
Gangrenous appendicitis	0.68 [0.25, 1.88]	0.456		
Perforated appendicitis	1.53 [0.44, 5.34]	0.505		

*Note:* ALT, alanine transaminase; AST, aspartate transaminase. Bold values indicates statistically significant differences (*p* < 0.05).

Abbreviations: CRP, C‐reactive protein; DB, direct bilirubin; TB, total bilirubin.

### 3.4. Comparisons of Out‐Of‐Bed Activity Rate (24 h Postoperatively) Between SNP and Non‐SNP Groups

Moreover, univariate logistic regression analysis illustrated that SNP management was linked to a higher rate of out‐of‐bed activity (24 h postoperatively) after LA surgery (OR = 2.39, 95% CI: 1.43–3.98). After the adjustment of the effects of CRP and postoperative 24‐h pain score, multivariate regression analysis indicated that SNP was still an independent factor for improving the out‐of‐bed activity rate after surgery (OR = 2.33, 95% CI: 1.37–3.96) (*p* < 0.05, Table 4).

**TABLE 4:** Univariable and multivariable analyses for out‐of‐bed activity rate (24 h postoperatively).

Items	Univariable		Multivariable	
	OR [95% CI]	*p* value	OR [95% CI]	*p* value
SNP	2.39 [1.43, 3.98]	**0.001**	2.33 [1.37, 3.96]	**0.002**
Gender	1.03 [0.62, 1.70]	0.919		
Age (year)	0.99 [0.98, 1.01]	0.458		
BMI	1.00 [0.94, 1.07]	0.892		
Fever peak (°C)	0.99 [0.87, 1.13]	0.865		
Leukocyte (10^9/L)	1.02 [0.97, 1.07]	0.432		
CRP	1.00 [1.00, 1.01]	**0.041**	1.01 [1.00, 1.01]	**0.026**
Neutrophil (%)	1.01 [0.99, 1.02]	0.46		
DB	0.99 [0.92, 1.07]	0.876		
TB	1.01 [0.98, 1.04]	0.457		
ALT	1.00 [0.99, 1.01]	0.678		
AST	1.00 [0.99, 1.02]	0.619		
VAS score (postoperative‐24‐h)	0.54 [0.29, 1.01]	0.054	0.70 [0.36, 1.35]	0.285
Diabetes	0.97 [0.38, 2.50]	0.956		
Hypertension	1.55 [0.62, 3.84]	0.347		
ASA score				
I	1.00 [Reference]			
II	0.94 [0.47, 1.89]	0.863		
Type				
Purulent appendicitis	1.00 [Reference]			
Gangrenous appendicitis	0.71 [0.24, 2.05]	0.522		
Perforated appendicitis	0.82 [0.22, 3.09]	0.764		

*Note:* ALT, alanine transaminase; AST, aspartate transaminase. Bold values indicates statistically significant differences (*p* < 0.05).

Abbreviations: CRP, C‐reactive protein; DB, direct bilirubin; TB, total bilirubin.

### 3.5. SNP Does Not Affect the Postoperative Drainage Tube Dislodged

In fact, a postoperative drainage tube dislodged has always been a serious clinical complication in LA. It has a negative impact on both the psychological feelings of patients and the clinical treatment effect. Univariate logistic regression analysis illustrated that SNP could decrease the incidence of postoperative drainage tube dislodged (OR = 0.34, 95% CI: 0.12–0.97) (*p* < 0.05, Table 5). However, after controlling the effects of postoperative 24‐h pain score and out‐of‐bed activity rate, SNP is no longer an independent factor in decreasing postoperative drainage tube dislodged (OR = 0.49, 95% CI: 0.16–1.51) (*p* > 0.05, Table 5).

**TABLE 5:** Univariable and multivariable analyses for drainage tube dislodged.

Items	Univariable		Multivariable	
	OR [95% CI]	*p* value	OR [95% CI]	*p* value
SNP	0.34 [0.12, 0.97]	**0.044**	0.49 [0.16, 1.51]	0.211
Gender	1.21 [0.44, 3.34]	0.716		
Age (year)	1.00 [0.97, 1.03]	0.962		
BMI	1.06 [0.92, 1.21]	0.426		
Fever peak (°C)	0.93 [0.51, 1.69]	0.812		
Leukocyte (10^9/L)	1.00 [0.93, 1.09]	0.908		
CRP	0.99 [0.99, 1.00]	0.28		
Neutrophil (%)	1.02 [0.98, 1.07]	0.358		
DB	1.08 [0.95, 1.24]	0.241		
TB	1.03 [0.99, 1.08]	0.127		
ALT	0.98 [0.94, 1.02]	0.29		
AST	0.95 [0.87, 1.03]	0.226		
VAS score (postoperative‐24‐h)	3.20 [1.13, 9.03]	**0.028**	2.26 [0.75, 6.77]	0.146
Out‐of‐bed activity rate (24 h postoperatively, %)	0.29 [0.11, 0.78]	**0.014**	0.36 [0.13, 1.01]	0.051
Drainage tube placement time (Day)	1.12 [0.73, 1.71]	0.612		
Diabetes	1.73 [0.38, 8.01]	0.481		
Hypertension	1.16 [0.26, 5.29]	0.845		
ASA score				
I	1.00 [Reference]			
II	1.90 [0.59, 6.06]	0.280		
Type				
Purulent appendicitis	1.00 [Reference]			
Gangrenous appendicitis	1.18 [0.15, 9.43]	0.876		

*Note:* ALT, alanine transaminase; AST, aspartate transaminase. Bold values indicates statistically significant differences (*p* < 0.05).

Abbreviations: CRP, C‐reactive protein; DB, direct bilirubin; TB, total bilirubin.

### 3.6. SNP Was Linked to the Patient’s Degree of Satisfaction After LA

From the perspective of clinical nursing effect, we finally explored the link between SNP management and patient satisfaction. Univariate logistic regression analysis illustrated that SNP significantly improved patients’ degree of satisfaction (OR = 15.47, 95% CI: 9.15–26.14). Even after controlling for postoperative 24 h pain score, out‐of‐bed activity (24 h postoperatively), drainage tube placement time (Day), drainage tube dislodged, and postextubation leakage, multivariate logistic regression analysis still suggested that SNP was an important factor in improving patients’ degree of satisfaction (OR = 16.02, 95% CI: 9.06–28.32) (*p* < 0.001, Table 6).

**TABLE 6:** Univariable and multivariable analyses for degree of satisfaction.

Items	Univariable		Multivariable	
	OR [95% CI]	*p* value	OR [95% CI]	*p* value
SNP	15.47 [9.15, 26.14]	**< 0.001**	16.02 [9.06, 28.32]	**< 0.001**
Gender	0.97 [0.63, 1.48]	0.881		
Age (year)	1.00 [0.99, 1.01]	0.794		
BMI	1.07 [1.01, 1.14]	0.021		
Fever peak (°C)	1.01 [0.89, 1.14]	0.92		
Leukocyte (10^9/L)	1.00 [0.96, 1.03]	0.815		
CRP	1.00 [1.00, 1.00]	0.455		
Neutrophil (%)	1.00 [0.98, 1.01]	0.563		
DB	0.96 [0.90, 1.02]	0.218		
TB	1.00 [0.97, 1.02]	0.822		
ALT	1.00 [1.00, 1.01]	0.588		
AST	1.00 [0.99, 1.01]	0.581		
VAS score (postoperative‐24‐h)	0.42 [0.23, 0.74]	0.003	1.04 [0.51, 2.12]	0.914
Out‐of‐bed activity rate (24 h postoperatively, %)	1.94 [1.18, 3.21]	0.009	1.27 [0.66, 2.45]	0.466
Drainage tube placement time (Day)	0.68 [0.56, 0.82]	**< 0.001**	0.62 [0.49, 0.79]	**< 0.001**
Drainage tube dislodged	0.26 [0.09, 0.75]	0.013	0.32 [0.08, 1.3]	0.112
Drainage tube leakage	0.66 [0.04, 10.62]	0.769		
Diabetes	0.81 [0.37, 1.79]	0.608		
Hypertension	1.71 [0.82, 3.56]	0.153		
ASA score				
I	1.00 [Reference]			
II	0.91 [0.50, 1.66]	0.767		
Type				
Purulent appendicitis	1.00 [Reference]			
Gangrenous appendicitis	1.02 [0.39, 2.71]	0.961		
Perforated appendicitis	0.65 [0.21, 2.06]	0.467		

*Note:* ALT, alanine transaminase; AST, aspartate transaminase. Bold values indicates statistically significant differences (*p* < 0.05).

Abbreviations: CRP, C‐reactive protein; DB, direct bilirubin; TB, total bilirubin.

## 4. Discussion

Appendectomy is the main technique for treating acute appendicitis. In the past, open surgery was a traditional method. With the construction of minimally invasive surgical methods, LA has been widely used in clinical practice [1]. About 7%–10% of individuals after appendectomy may experience infection‐related complications, including wound infection, abdominal abscess, and postoperative intestinal obstruction [2]. Despite the controversy, AD remains an important diagnostic and therapeutic approach [4, 15]. While, if not maintained properly, it may trigger various complications such as unplanned detachment, blockage, distortion, rupture, and damage, resulting in extended hospitalization and increased discomfort, medical costs, and death rates for patients after surgery [2, 3].

Herein, a retrospective analysis of the clinical parameters of drainage tubes was conducted after LA in individuals with SNP‐managed acute appendicitis. Although acute appendicitis is a prevalent acute abdomen, and minimally invasive LA surgery has been widely used, the placement of postoperative AD tubes is still a common way to observe postoperative abdominal complications. However, the misplacement of the drainage tube or poor drainage due to various reasons may delay the adjustment of the disease diagnosis and treatment strategy and lead to more serious complications [2]. Meanwhile, the development of SNPs for AD tubes has not yet been proposed. Therefore, our team has developed this SNP management for AD tubes based on common clinical nursing problems. Through this process, nursing staff are encouraged to provide more effective feedback on the nursing management of drainage tubes after LA. The analysis of the results showed that the differences were of clinical significance, and SNPs were associated with indicators of improved nursing efficiency, such as adjusted drainage tube strategies, and correlated with enhanced recovery metrics and patient satisfaction. In the high turnover and time‐sensitive context of an emergency surgical service, where patient conditions and nursing workloads can vary widely, such a standardized, protocol‐driven approach may offer particular value by reducing practice variation and ensuring consistent, high‐quality postoperative monitoring for this common emergency condition.

In fact, there is a focus on whether to place an AD tube after LA for a long time. Previous literature reported that it was still unclear whether prophylactic placement of AD tubes can reduce the risk of abdominal abscess formation after LA in individuals with perforated appendicitis [8, 16]. Meta‐analyses have shown that the prophylactic use of AD tubes after complicated appendectomy does not benefit patients, especially in children. On the contrary, it elevates the occurrence of associated complications, such as longer postoperative hospitalization and higher overall occurrence of postoperative complications and wound infection [2]. Another retrospective study suggested that AD appears to have no additional benefit for perforated appendicitis but may prolong hospital stay after LA [17]. Other studies have used a comparative model of laparoscopic and open appendectomy, with similar results to the cohort study of LA surgery for acute appendicitis, which was difficult to perform under full laparoscopy [17–19].

Even though the placement of a peritoneal drain after LA is still an observational strategy for preventing complications [3, 4, 8]. Some investigations have shown that the positioning of abdominal drains is related to an elevated risk of wound infection, potentially attributable to the inclusion of open appendectomy in the cohort study [20, 21]. Compared with laparoscopic examination, open appendectomy usually has a higher incidence of wound infection [22–24]. However, it has also been reported that placement of an abdominal drain after LA could decrease the occurrence of postoperative intra‐abdominal abscess complications after perforated appendectomy (6.0% vs. 20.0%, *p* = 0.05) [25]. The patients selected for our queue are all those who underwent LA surgery. Although the degree of appendicitis was uneven (including acute noncomplicated appendicitis and complicated appendicitis), the surgical approach was uniform. Moreover, this study focused on nursing process management, and there have been no large‐scale cohort studies reporting similar research on the exploration of standardized management models for drainage tubes. Although we manage drainage tubes from a nursing perspective, the focus is not on the necessity of drainage. However, for LA patients with drainage tubes, SNP may provide better management and nursing care. Our results suggest potential clinical benefits that warrant further investigation in similar patient populations.

Recently, research reported that, especially for acute appendicitis with a higher grade (grade ≥ II), AD after appendectomy has the advantage of shortening the period of postoperative antibiotic use and hospital stay [26]. Another study has examined the effects of several types of drainage tubes on postoperative complications, indicating that active drainage may be more effective in removing peritoneal effusion and reducing the occurrence of abdominal abscesses [27]. For these patients, the nursing management of AD is critical. Herein, a retrospective cohort investigation was conducted to analyze the data of individuals with postoperative drainage tube placement for acute appendicitis and to compare the clinical effects of process‐based nursing management on AD tubes. The results of this study showed that SNPs could effectively improve the postoperative recovery of patients and benefit them. Based on this, we plan to further conduct a multicenter cohort study to summarize the experience and promote the nursing management of patients with postoperative AD tubes, benefiting more patients, not just limited to acute appendicitis. If validated, the implementation of a standardized nursing protocol could help streamline postoperative care pathways for emergency surgical patients, contributing to more efficient resource utilization and more predictable recovery trajectories within busy clinical settings.

However, this study has several limitations. First, the single‐center, pre–post cohort design, while clarifying the temporal implementation of the SNP, remains susceptible to inherent historical biases that cannot be fully eliminated. For instance, unmeasured temporal factors such as healthcare environment improvements or shifting patient expectations may still bias the results, even with unchanged surgical teams and protocols. Second, the decision to place an AD tube was surgeon‐dependent, but baseline characteristics and appendicitis severity were comparable between groups, and confounding factors were corrected by multivariate regression. Third, the exclusion of elderly patients and those lost to follow‐up may affect the representativeness and generalizability of the cohort. Fourth, surgical timing was variable, all procedures were performed emergently after diagnosis, and anesthesia personnel rotations could introduce variability in anesthesia‐related outcomes. Future prospective, randomized, or multicenter studies are warranted to validate the efficacy of this SNP and address these limitations.

## 5. Conclusions

Although laparoscopic minimally invasive surgery is now the main treatment for acute appendicitis, the use of AD tubes remains controversial. However, drainage is still an important measure for clinical monitoring and complication prevention. This investigation evaluated for the first time the influence of standardized nursing care for AD tubes after LA surgery for acute appendicitis. Our study found that the implementation of a standardized nursing protocol for AD tubes was associated with reduced postoperative pain, earlier mobilization, and improved patient satisfaction among patients undergoing LA. Although these findings are promising and underscore the potential value of structured nursing interventions, they are derived from a single‐center, retrospective design. Further prospective studies are warranted to validate these associations and establish causal relationships before broader clinical implementation can be recommended.

## Author Contributions

Chaobo Chen and Zipeng Xu: conceptualization and writing–original draft. Qinyan Yang, Yanan Zhou, and Genxi Tong: investigation and review. Jiamei Lin and Fengjun Cai: mathematical design. Wenhui Shi and Yan Huang: case organization, data collection, and analysis.

## Funding

This work was supported by the Top Talent Support Program for Young and Middle‐Aged Professionals of the Wuxi Health Committee (HB2023116) and the Appropriate Technology Promotion Project of the Wuxi Health Commission (T202432).

## Disclosure

All authors reviewed and gave their approval to the final version.

## Ethics Statement

The Ethics Committee of Xishan People’s Hospital of Wuxi City waived the necessity of written informed consent because of the study’s retrospective design (No. xs2025ky001). The investigation was conducted as per the Declaration of Helsinki.

## Conflicts of Interest

The authors declare no conflicts of interest.

## Data Availability

The data that support the findings of this study are available on request from the corresponding author (Chaobo Chen). The data are not publicly available due to privacy or ethical restrictions.
